# Exploring Symptoms of Post-traumatic Stress Disorders and Perceived Social Support among Patients with Burn Injury

**DOI:** 10.7759/cureus.2669

**Published:** 2018-05-22

**Authors:** Abeer Ashfaq, Usman G Lashari, Saad Saleem, Sadiq Naveed, Hafsa Meraj, Ahmed Waqas

**Affiliations:** 1 Medicine, CMH Lahore Medical and Dental College, Lahore Cantt, Pakistan; 2 Family Medicine, United Arab Emirates University, Al-Ain, Abu Dhabi; 3 Psychiatry, KVC Hospitals, Kansas City, USA; 4 Medicine, Sharif Medical and Dental College, Jati Umra, Lahore, PAK; 5 Department of Psychiatry, CMH Lahore Medical College and Institute of Dentistry

**Keywords:** ptsd, burn, lahore, pakistan, social support, trauma, post-traumatic stress disorder

## Abstract

Introduction

Burns are a serious public health problem globally, causing an estimated 265,000 deaths per year. Although the association of burn injuries with mortality and morbidity rates has been well established, data on their psychological consequences are scarce. The present study explores the frequency of post-traumatic stress disorder (PTSD) and perceived social support among patients with burn injuries in Pakistan.

Methods

This cross-sectional study was conducted at two teaching hospitals in Lahore, Pakistan from May 2015 to July 2015. Eighty patients with burn injuries were included by convenience sampling and interviewed with a specifically designed questionnaire with items on demographics, and the Impact of Events Scale-Revised (IES-R) and Multidimensional Scale of Perceived Social Support (MSPSS) instruments.

Results

Data were analyzed for a total of 80 participants: 56 women (70.0%) and 24 men (30.0%). Mean age was 35.74 (11.15) years. A high proportion of participants perceived highest social support from friends, reported high ego resiliency levels, had more severe symptoms of avoidance and intrusion, and had high overall PTSD scores. There were no differences between groups in the proportions of respondents who reported high perceived social support from significant others or family, overall social support or symptoms of hyperarousal.

Conclusion

The findings reflect a high frequency of PTSD symptomatology and poor social support among Pakistani patients with burn injuries in our sample. These factors can exacerbate the patient’s physical injury, delaying both their physical and mental rehabilitation.

## Introduction

A burn is empirically defined as a wound inflicted on the skin and underlying structures during exposure to incendiary or unstable materials; however, it also has psychosocial implications [1]. Burns are a serious global health problem, with an estimated 265,000 deaths reported each year [2]. Although the association of burn injuries with mortality and morbidity rates has been well established, data on their psychological consequences are scarce in developing countries.

In comparison to developed countries, mortality and physical and mental morbidity associated with burn injuries pose a greater concern in the developing world. In Pakistan, for example, cases of stove burns, domestic abuse, and accidental burns are on the rise. Accidents and domestic issues are persistently cited as the major reasons for burn injuries in Pakistan [3]. These practices threaten the social fabric, and also inflict mental suffering on the individual owing to low self-esteem, poor body image, and social stigma [4, 5]. These factors also lead to the genesis of psychiatric illnesses among these patients. In fact, a number of studies have identified a very high incidence of psychopathologies such as depression, sleep disturbances, sexual dysfunction, poor marital relationships, social and generalized anxiety, substance dependence, post-traumatic stress disorder (PTSD) and agoraphobia among patients with burn injuries [4-6].

Studies report that as many as one-fifth of the people who have suffered significant burn injury later experience symptoms of PTSD [7]. These disturbing symptoms include nightmares, upsetting ideations, distress, avoidance, sleep disturbances and associated flashbacks [8]. Waqas et al. reported a very high prevalence of PTSD among burn patients, with a prevalence rate as high as 69% [9]. The high incidence and severity of PTSD have been associated with extensive post-burn scarring, female gender, large burn surface area, pretraumatic depressive behaviors, low psychological resilience, and inadequate social support [9]. However, those who are provided with emotional help and mitigation are less likely to develop symptoms of PTSD [9].

Among patients with burn injuries, social support has been identified as a major buffer of stress that intensifies the healing process, as well as shortening the resuscitation phase and duration of post-traumatic recovery [10]. This emphasizes the fact that care for burn patients should not only focus on treating their wounds, but should also involve families, significant others, and friends in a more holistic approach [3]. Unfortunately, due to lack of awareness and limitations in education, effective counseling and psychosocial interventions, these patients report poor social support in Pakistan [10]. Surprisingly, the effects of poor social support translate at the molecular level, leading to a substantial decrease in the level of interleukins 1 and 8 in the body, which are crucial for recovery among burn patients [11].

The paucity of data regarding the frequency of PTSD along with poor social support levels in Pakistan warranted this study. It was accordingly designed to elucidate the frequency of PTSD, the structure of social support, and their correlates among patients with burn injury in Lahore, Pakistan.

## Materials and methods

This descriptive cross-sectional study was conducted at two teaching hospitals in Lahore, Pakistan from May 2015 to July 2015. Three final-year medical students were recruited in a convenience sample of 80 burn patients presenting at different departments of these hospitals for follow-up clinical consultation. Prior to the start of this study, the ethical basis of this study was reviewed by the Ethical Review Board of CMH Lahore Medical College and Institute of Dentistry, Lahore Cantt, Pakistan. All participants were briefed about the objectives of the study, and were ensured anonymity and that no individual-level findings would be reported.

These participants were interviewed in Urdu by three interviewers with a specifically designed questionnaire. The instrument comprised demographic items, and the Urdu translations of the Impact of Events-Revised Scale (IES-R) and the Multidimensional Scale of Perceived Social Support (MSPSS).

The Urdu translation of the MSPSS was used to evaluate the levels of perceived social support among participants [12]. This version has been found to be valid and reliable among Pakistani women from Rawalpindi district, Pakistan [12]. It comprises 12 items which are rated by respondents on a 7-point Likert scale. It has excellent internal consistency (Cronbach’s alpha = 0.92) and a unidimensional factorial structure [12]. A total score ranging from 12 to 84 is obtained by summing the scores for each item, with higher scores corresponding to greater levels of social support [12]. It also yields scores on three subscales: friends, family and significant others [12].

The Urdu language version of the IES-R has shown excellent reliability (Cronbach’s alpha = 0.94) and construct validity in Pakistan [13]. It consists of 22 items yielding scores for three clusters of symptoms including intrusion, avoidance, and hyperarousal. It yields a total score ranging from 0 to 88, with a score of 33 taken as a cut-off for screening to detect cases of PTSD [14,15].

All data were analyzed in SPSS v. 20 (IBM, Chicago, IL, USA). Descriptive statistics were run for categorical and quantitative variables. The chi-squared test of proportions was used to identify significant differences in the proportions of symptom clusters of PTSD and levels of perceived social support. Pearson’s correlation coefficient was calculated to analyze associations between PTSD levels and scores on the MSPSS subscales. A t-test for independent samples was used to determine the significance of differences in mean IES-R and MSPSS scores among different groups of respondents.

## Results

Data were analyzed for a total of 80 participants: 56 women (70.0%) and 24 men (30.0%). Mean age was 35.74 (11.15) years. Most participants were married (n = 57, 71.20%), and had a monthly household income greater than PKR 33,000 (approximately equivalent to USD 285.29). Slightly more than half of the participants were non-Punjabi (n = 43, 53.80%); most self-identified as Muslims (n = 77, 96.2%), were literate (n = 58, 72.5%), and lived in urban areas (n = 66, 82.5%). Detailed demographic data are presented in Table 1.

**Table 1:** Demographic characteristics of burn patients (n = 80).

Variable	Subcategory	Frequency (n)	Percentage (%)
Gender	Male	24	30.0
	Female	56	70.0
Relationship status	Single	19	23.8
	Married	57	71.2
	Divorced	3	3.8
	Separated	1	1.2
Household income	Low	36	45.0
	High	44	55.0
Ethnicity	Punjabi	37	46.2
	Non-Punjabi	43	53.8
Religion	Islam	77	96.2
	Other	3	3.8
Education	Illiterate	22	27.5
	Literate	58	72.5
Background	Rural	14	17.5
	Urban	66	82.5

Only two participants (2.50%) had received cosmetic treatment, and 11 (13.60%) had permanent physical deformities. A total of seven (8.60%) reported experiencing other major traumas in life including loss of a parent or child, or exposure to natural disasters. A small proportion of respondents reported having experienced relationship problems (n = 5, 6.2%) and domestic abuse (2, 2.5%). A total of three (3.7%) reported suicidal ideation, nine (11.10%) had burns on their face, and 76 (93.8%) had burns on other parts of their body.

Among all the participants the mean IES-R score was 38.61 (11.27). A mean score of 1.75 (0.54) was found for the avoidance subscale, 1.78 (0.53) for the intrusion subscale, and 1.73 (0.58) for the hyperarousal subscale. On the MSPSS scale, the mean total score was 4.14 (0.56). Subscale analysis disclosed a mean score of 4.06 (0.56) for perceived social support from significant others, 4.14 (0.64) from family, and 4.22 (0.61) from friends.

Overall, 63.7% of the respondents were considered positive for symptoms of PTSD; 67.5% scored positive for avoidance, 65.0% for intrusion, and 45.0% for hyperarousal. According to the MSPSS score, 48.8% of the respondents reported adequate social support overall, while 38.8% perceived adequate social support levels from family, 73.8% from friends, and 46.2% from significant others.

According to the chi-squared statistic, a significantly higher proportion of participants perceived higher social support from friends, ego resiliency levels, and greater severity of symptoms of avoidance and intrusion. No statistically significant differences were seen in the proportions of respondents above and below the cut-off values for perceived social support from significant others or from family, overall social support, or symptoms of hyperarousal (Table 2).

**Table 2:** Frequency of burn patients reporting high PTSD symptomatology and poor social support (n = 80). ^1^ denotes P < 0.001, ^2^ denotes P < 0.01, ^3^ denotes P < 0.05, and ^4^ denotes P > 0.05. PTSD: Post-traumatic stress disorder

Variable	Category	Frequency (n)	Percentage %	Chi squared
Significant other	≤4.00	43	53.8	43.00^4^
	+4.01	37	46.2	
Family	≤4.25	49	61.3	49.00^4^
	+4.26	31	38.8	
Friends	≤3.75	21	26.2	21.00^1^
	+3.76	59	73.8	
Social support	≤4.25	41	51.2	41.00^4^
	+4.26	39	48.8	
Ego resilience	≤2.64	19	23.8	19.00^1^
	+2.65	61	76.2	
Avoidance	≤1.38	26	32.5	54.00^2^
	+1.39	54	67.5	
Intrusion	≤1.50	28	35.0	52.00^2^
	+1.51	52	65.0	
Hyper-arousal	≤1.67	44	55.0	36.00^4^
	+1.68	36	45.0	
PTSD	≤33.00	29	36.2	51.00^3^
	+34.00	51	63.7	

Only one (1.3%) participant reported being diagnosed with a mental illness, receiving counseling sessions and group therapy. A total of 24 participants reported using illicit substances including tobacco (n = 18, 22.5%), sleeping pills (n = 3, 3.75%), antidepressants (n = 2, 2.5%) or cocaine (n = 1, 1.3%). Several reasons were cited for starting substance abuse, most frequently peer pressure (n = 18, 22.5%), depression (n = 1, 1.3%), and stress (n = 1, 1.3%). Among these participants, only five (6.25) wished to quit their substance abuse.

Independent sample t-tests showed that participants who reported a higher income perceived a better overall level of social support, and those from a rural background had fewer PTSD symptoms. No group differences in PTSD or social support scores were found among participants of different genders, partner relationship categories, religions or levels of education (Table 3).

**Table 3:** Group differences in PTSD scores and social support (n = 80). ^1 ^denotes P < 0.001, * denotes P > 0.05. PTSD: Post-traumatic stress disorder

Variable	Subcategory	PTSD		t-statistic	Social support		t-statistic
		Mean	SD		Mean	SD	
Gender	Male	37.08	11.57	-0.79*	4.21	0.37	0.69*
	Female	39.27	11.18		4.11	0.63	
Relationship	Single	38.79	13.91	0.78*	4.04	0.93	0.56*
	Other	38.56	10.44		4.17	0.39	
Income	Low	39.00	13.29	0.79*	3.96	0.76	-2.68^1^
	High	38.30	9.44		4.29	0.25	
Ethnicity	Punjabi	36.89	13.12	-1.24*	4.15	0.65	0.07*
	Other	40.09	9.29		4.14	0.48	
Religion	Islam	38.57	11.42	-1.16*	4.15	0.57	0.62*
	Other	39.67	7.77		3.94	0.32	
Education	Illiterate	36.64	12.99	-0.97*	4.04	0.50	-0.98*
	Literate	39.36	10.57		4.18	0.58	
Background	Rural	30.21	12.48	-3.25^1^	4.13	1.00	-0.05*
	Urban	40.39	10.23		4.14	0.43	

There was no significant correlation between subscale scores on the IES-R and MSPSS (Table 4).

**Table 4:** Pearson correlation (r) between PTSD symptoms and social support (n = 80). PTSD: Post-traumatic stress disorder; MSPSS: Multidimensional Scale of Perceived Social Support.

Social support	PTSD	Avoidance	Intrusion	Hyper arousal
Significant other	0.057	0.159	0.051	-0.076
Family	-0.041	0.029	0.015	-0.189
Friends	0.042	0.116	0.060	-0.082
Overall MSPSS score	0.019	0.106	0.045	-0.127

## Discussion

The present study found a high frequency of PTSD and poor social support among Pakistani patients in Lahore with burn injuries. A high proportion of patients reported using illicit substances and receiving poor social support, while a few also reported problematic marital relationships and suicidal ideation.

Our results are consistent with another multicenter study in Pakistan that reported a prevalence of PTSD of 69% among burn patients [9]. Surprisingly, these figures from Pakistan are significantly higher than those in western countries [16, 17]. For example, the global prevalence of PTSD among burn patients has been reported to be as high as 45%, with rates of 21.2% in the USA, 2.2% in Finland, 33% in The Netherlands, and 13% in Germany [17, 18]. A multitude of causes may be responsible for the high psychiatric morbidity among Pakistani patients with burn injuries, including poor socioeconomic conditions and social support networks, public and self-stigma, and lack of awareness [10, 19].

Among the symptoms of PTSD, the most frequently reported were avoidance and intrusion, and the least frequently reported symptom was hyperarousal. These results are consistent with another study of the prevalence of PTSD among healthcare providers in Gaza after Israeli attacks [20]. Symptoms of intrusion entail the unexpected recurrence of traumatic memories (flashbacks), thus causing the individual to re-experience the same emotions as those attached to their initial injury [20]. In contrast, avoidance symptoms are generally associated with emotional numbing due to trauma, leading to repressed emotions, anger and grief, and sometimes eventually leading individuals to sever their relationships with society to avoid re-experiencing stimuli related to their trauma [21]. Hyperarousal includes a cluster of symptoms such as sleeping problems, anger outbursts, anxiety, panic, self-destructive behaviors, and feelings of guilt and shame [22].

A high proportion of respondents in the present study reported substance use following their traumatic experience with burn injuries. A high incidence of substance use is also associated with higher rates of PTSD [19]. Giannoni-Pastor et al. reported a weak to moderate association of PTSD symptoms with substance use disorders and alcohol use disorders in their meta-analysis [19]. Hence, the use of illicit substances adds further challenges to the existing psychological injury, and predisposes these patients to psychiatric comorbidities.

The Pakistani burn patients in the present study reported receiving higher levels of social support from their friends than from their families and significant others. These findings contrast with an earlier study in Pakistan that reported family structure to make the largest contribution to burn patients’ social support network [10]. The formation of a strong social support network among burn patients is governed by an interplay of social, psychological and biological factors. According to Waqas et al., higher social support among Pakistani patients with burn injuries is associated with male gender, Punjabi ethnicity, higher levels of education, larger burn surface area, cosmetic surgery, and psychological resilience [10].

Our analysis revealed a significant association between high-income levels and better social support. This finding can be explained by a multitude of factors; for example, patients belonging to higher socioeconomic classes might have access to better burn management programs, mental health services and resources to leverage strong social support networks [10]. Moreover, with better access to healthcare, cosmetic surgical treatments can be sought by these groups, thus leading to an improved sense of wellbeing, and greater social support from the family and society [10].

Despite its limitations, this study has several strengths. Most importantly, it addresses the paucity of data on the relationship between burn injuries and PTSD in Pakistan. But because this study explored PTSD and social support in a small sample of patients in one city in Pakistan, the results should not be generalized to the entire Pakistani population. Because of the small sample size, analyses to detect possible group differences could not be done. Moreover, the cross-sectional design of this study does not make it possible to establish causal relationships between variables.

The results of this study can nonetheless help guide the direction and design of future epidemiological studies and psychosocial interventions aimed at improving the psychological health of patients with burn injuries.

## Conclusions

A high frequency of PTSD symptomatology and poor social support were found in our sample of Pakistani patients with burn injuries. A high percentage of patients reported using illicit substances, and a few also reported problematic marital relationships and suicidal ideation. These factors add further challenges to those created by the patient’s physical injury, delaying both their physical and mental rehabilitation. Therefore, the clinical management of patients with burn injuries should be based on an interdisciplinary approach to ensure a high quality of clinical, psychological and social care.
